# The Zn-finger of *Saccharomyces cerevisiae* Rad18 and its adjacent region mediate interaction with Rad5

**DOI:** 10.1093/g3journal/jkab041

**Published:** 2021-02-11

**Authors:** Orsolya Frittmann, Vamsi K Gali, Miklos Halmai, Robert Toth, Zsuzsanna Gyorfy, Eva Balint, Ildiko Unk

**Affiliations:** 1 Biological Research Centre, Szeged, Eotvos Loránd Research Network, The Institute of Genetics, Szeged, H-6726, Hungary; 2 Doctoral School of Biology, University of Szeged, Szeged, H-6720, Hungary

**Keywords:** Rad18-Rad5 interaction, yeast genetics, DNA damage tolerance, yeast two-hybrid

## Abstract

DNA damages that hinder the movement of the replication complex can ultimately lead to cell death. To avoid that, cells possess several DNA damage bypass mechanisms. The Rad18 ubiquitin ligase controls error-free and mutagenic pathways that help the replication complex to bypass DNA lesions by monoubiquitylating PCNA at stalled replication forks. In *Saccharomyces cerevisiae*, two of the Rad18 governed pathways are activated by monoubiquitylated PCNA and they involve translesion synthesis polymerases, whereas a third pathway needs subsequent polyubiquitylation of the same PCNA residue by another ubiquitin ligase the Rad5 protein, and it employs template switching. The goal of this study was to dissect the regulatory role of the multidomain Rad18 in DNA damage bypass using a structure-function based approach. Investigating deletion and point mutant *RAD18* variants in yeast genetic and yeast two-hybrid assays we show that the Zn-finger of Rad18 mediates its interaction with Rad5, and the N-terminal adjacent region is also necessary for Rad5 binding. Moreover, results of the yeast two-hybrid and *in vivo* ubiquitylation experiments raise the possibility that direct interaction between Rad18 and Rad5 might not be necessary for the function of the Rad5 dependent pathway. The presented data also reveal that yeast Rad18 uses different domains to mediate its association with itself and with Rad5. Our results contribute to better understanding of the complex machinery of DNA damage bypass pathways.

## Introduction

Due to the continuous exposure to environmental agents that can harm or modify the inheriting material, cells are equipped with several DNA repair mechanisms that preserve the integrity of DNA by removing any alterations and restoring the original sequence. Some lesions, however, escape repair and pose an obstacle to the moving replication fork. Cells avoid the fatal consequences of stalled replication by activating the so-called DNA damage tolerance (DDT) mechanisms that facilitate replication through DNA lesions without removing them. DDT can be error-free or error-prone depending on the type of damage and the mechanism of bypass, and accordingly, it can contribute to genomic stability or it can destabilize the genome by introducing mutations during the bypass process.

In the yeast *Saccharomyces cerevisiae*, the Rad6/Rad18 complex governs at least three subpathways of DDT on UV-damaged DNA (Torres-Ramos *et al.* 2002). Specialized translesion synthesis (TLS) DNA polymerases constitute the *REV1-*dependent error-prone subpathway together with the Def1 protein. The TLS polymerases of this branch, the Rev1 protein and the Rev3-Rev7 formed DNA polymerase ζ, either introduce incorrect nucleotides during lesion bypass with high frequency or extend from it, respectively, resulting in mutagenesis (Lawrence and Maher 2001). The Def1 protein enables the exchange between the replicative and TLS polymerases by promoting proteasomal degradation of the catalytic subunit of the stalled replicative polymerase (Daraba *et al.* 2014). The second branch consists of the *RAD30* encoded TLS DNA polymerase η that is uniquely error-free opposite pyrimidine dimers, the most frequent UV-induced DNA lesions (McDonald *et al.*1997; Johnson *et al.*1999). The third branch involves the *RAD5*, the *MMS2*, and the *UBC13* genes, and it operates through template switching using the newly synthesized strand of the sister chromatid as a template resulting in error-free bypass (Johnson *et al.*1992; Broomfield *et al.*1998; Hofmann and Pickart 1999; Zhang and Lawrence 2005; Blastyák *et al.* 2007). The two subbranches involving TLS polymerases are activated by PCNA monoubiquitylation at lysine 164 (K164) by Rad6/Rad18, whereas the *MMS2* branch requires PCNA polyubiquitylation at the same residue performed by Rad5 together with Mms2 and Ubc13 (Hoege *et al.* 2002; Stelter and Ulrich 2003). In the polyubiquitylation reaction, the Mms2/Ubc13 complex acts as an ubiquitin conjugase, and Rad5 acts as an ubiquitin ligase (Hofmann and Pickart 1999).

Rad18 together with Rad6 controls an early step in the activation of DNA damage bypass mechanisms by forming an ubiquitin conjugase/ligase complex that monoubiquitylates PCNA at DNA damage-stalled replication complexes (Bailly *et al.*1994; Hoege *et al.* 2002). In the complex, a homodimer Rad18 binds a single Rad6 (Huang *et al.* 2011; Masuda *et al.* 2012). The ubiquitin conjugase Rad6 is a small protein, but the ubiquitin ligase Rad18 has several domains. At the N-terminus, Rad18 contains a RING domain characteristic of a group of E3 ubiquitin ligases, which is essential for its function (Tateishi *et al.* 2000). In humans it was found to contribute to Rad6 binding and to mediate homodimerization (Tateishi *et al.* 2000; Huang *et al.* 2011; Masuda *et al.* 2012). In the middle of the protein, there is a Zn-finger the exact function of which is still debated. The Zn-finger of human Rad18 was shown to bind ubiquitin, even the crystal structure of their complex was resolved (Bish and Myers 2007; Notenboom *et al.* 2007; Huang *et al.* 2009; Rizzo *et al.* 2014). However, another study found that the Zn-finger mediates the association of human Rad18 with itself, as the C207F amino acid change affecting one of the core cysteine residues of the Zn-finger prevented homodimerization (Miyase *et al.* 2005). Moreover, HLTF and SHPRH the two human homologs of Rad5 were shown to bind Rad18 through its Zn-finger (Unk *et al.* 2006, 2008; Zeman et al. 2014). The SAP (SAF-A/B, Acinus and Pias) domain was identified as a DNA binding domain, and it was found to have the same role in human Rad18 (Aravind and Koonin 2000; Notenboom *et al.* 2007; Tsuji *et al.* 2008). Close to the C-terminus Rad18 has a sequence responsible for Rad6 binding (Bailly *et al.*1997b; Watanabe *et al.* 2004; Notenboom *et al.* 2007). Both DNA and Rad6 binding are essential for the role of Rad18 in DDT. Beside these, two small motifs have been identified in the Rad18 sequence. One is a nucleotide binding motif situated N-terminal to the Rad6 binding site, the so-called Walker type A box characteristic to nucleotide binding proteins, and indeed, Rad18 has a single stranded DNA dependent ATP-ase activity (Bailly *et al.*1994, 1997a). The other is a SUMO interacting motif between the RING and Zn-finger domains, which confers higher ubiquitylating activity toward sumoylated PCNA (Parker and Ulrich 2012).

In spite of the accumulating data, the exact choreography of Rad18 action in DDT is elusive since the functions of some of its known domains are still debated and specific parts of the protein mediating its several interactions have not been identified. In this study, we set out to investigate how regions in Rad18 lacking well-defined domains contribute to its role in DNA damage tolerance. Based on our results, we could assign a novel function to the Zn-finger motif of yeast Rad18 and refine the current model of DDT.

## Materials and methods

### Yeast strains and plasmids

Yeast strains used in the genetic studies are derivatives of EMY74.7 (*MAT*a, *his3*-Δ*1*, *leu2-3*, -*112*, *trp1*Δ, and *ura3-52*). Deletions were constructed by gene replacement. For ectopic complementation of the *RAD18* deletion, the wild-type (wt) and the mutant variants of *RAD18* with their promoter and terminator sequences, from −600 to 300 nucleotides (nt) after the STOP codon, were cloned into the yeast centromeric vectors YCplac33 and YCplac111 having the *URA3* and *LEU2* marker genes, respectively. The C-terminal deletions of *RAD18* (C1–C3) were constructed by replacing the XbaI-StuI fragment of the wt *RAD18* with XbaI-StuI flanked PCR products having the shortened sequences and a STOP codon at the desired position. For the N-terminal deletions (N1, N2), the MscI-NdeI fragment of the wt *RAD18* was exchanged to MscI-NdeI flanked PCR products containing the shortened sequences. The CC190,193GG point mutations in *RAD18* were generated by a PCR based method according to the Stratagene “Quick Change Site Directed Mutagenesis” protocol. The wt and the mutant *RAD18* genes in the YCplac33 vector were C-terminally tagged with three copies of the hemagglutinin epitope tag (3-HA) by a PCR-based strategy (Knop *et al.*1999) in EMY74.7, previously made *rad18Δ*. Strains carrying Rad18 expressing constructs were always grown in synthetic dropout media to maintain the plasmids. All mutations were confirmed by sequencing.

For the two-hybrid assays, the yeast strain PJ69-4A (*MAT***a** *trp1-901 leu2-3,112 ura3-52 his3-200 gal4Δ gal80Δ MET2: GAL7-lacZ LYS2: GAL1-HIS3 GAL2-ADE2*) (James *et al.*1996), and its *rad18Δpol30-K164R* variant were used, where the wt*POL30* gene at the genomic locus was replaced by a mutant coding for the K164R mutant PCNA. The whole coding regions of the wt and mutant *RAD18*, *RAD5*, and *RAD6* genes were cloned into the Gal4-BD vector pGBT9 + 1 resulting in pID685 (wt Rad18), pID771 (N1), pID704 (N2), pID1047 (Zn*) pID623 (Rad5), and pID231(Rad6). The wt and mutant *RAD18*, the *RAD5*, and the *POL30* genes were cloned into the Gal4 activation domain fusion vector pGAD424 + 1 resulting in pID684 (wt Rad18), pID768 (N1), pID700 (N2), pID1046 (Zn*), pID145 (Rad5), and pID999 (Pol30) plasmids. A region of *RAD18* coding for amino acids 170–230 consisting the wt, or CC190,193GG mutant Zn-finger was cloned into pGBT9 + 1 resulting in plasmids pID1149 (Znf+p), and pID1150 (Znf-p), respectively.

Detection of *in vivo* PCNA ubiquitylation was carried out in a DF5a strain (*MATa, trp1-1, his3–200, ura3-52, lys2-801*, and*leu2-3,2-112*) (Finley *et al.*1987) that was made *rad18Δ* and *pol30Δ*, and expressed an N-terminally tagged 7His-PCNA under its own promoter as the sole source of PCNA from the YCplac33 plasmid.

### Sensitivity assays

For qualitative assays, *rad18Δ*cells carrying the *RAD18* gene or its mutant derivatives on a plasmid were grown in synthetic complete media lacking uracil (SC-ura) overnight at 30 °C. Cells were counted using a Bürker chamber, then the cultures were adjusted to the same density and a series of 10-fold dilutions of the cultures were spotted onto SC-ura plates. For UV sensitivity, the plates were exposed to different doses of UV (254 nm) radiation and incubated at 30 °C in the dark for 3 days. Methyl methanesulfonate (MMS), bleomycin and hydroxyurea (HU) sensitivities were assayed on plates supplemented with the indicated amount of the chemicals (Sigma-Aldrich).

For quantification of UV sensitivity, cells were spread onto SC-ura plates at appropriate dilutions and irradiated with UV for given times to apply the required dosages. Colonies were counted after incubating the plates in the dark at 30 °C for 3–5 days.

### UV-induced and spontaneous mutation rates

UV irradiation induced forward mutation frequencies at the *CAN1* locus were measured by comparing the numbers of *can1^R^*colonies at different UV doses, selected on SC-leu/arg plates containing canavanine, with the number of colonies on SC-leu plates exposed to the same UV doses. Spontaneous forward mutation frequencies were measured also at the *CAN1* locus. In parallel, ten single colonies for each strain were diluted in water, and approximately 10 cells from each colony were inoculated into 500 µl SC-leu in a microtiter plate and grown for 3 days at 30 °C. The cultures were plated onto canavanine containing SC-leu/arg plates, and the number of mutants in case of each culture was counted for 10^7^ plated cells. Mutation frequencies for each strain were determined using the median value of mutations of the ten cultures and a chart based on the Lea-Coulson fluctuation model. The mean values of the median mutation frequencies and standard deviations were calculated from five experiments.

### Yeast two-hybrid

The binding and activation domain fusion constructs were co-transformed into the wt or the modified PJ69-4A strains, and transformants were selected on SC-leu/trp medium. Single colonies of the transformants were resuspended in water at equal densities and spotted onto SC-leu/trp plates to demonstrate equal growth, and onto SC-leu/trp/his plates to assess the *HIS3* gene expression that reflects protein-protein interaction. Where indicated, plates were supplemented with the given doses of 3-amino-1,2,4-triazole to test the strength of the interactions. Plates were incubated at 30 °C for 3–6 days.

### Protein techniques and antibodies

To show the level of Rad18 gene expression, 10 ml yeast cultures grown in SC-ura were harvested at A_600_:1. Whole cell extracts were prepared by glass-bead lysis method in 1XPBS (137 mM NaCl, 2.7 mM KCl, 10 mM Na_2_HPO_4_, and 1.8 mM KH_2_PO_4_)supplemented with 1 mM EDTA, 10% glycerol, and protease inhibitors. Cell lysates were quantified by Bradford. Equal amounts of whole cell lysates were separated by SDS-PAGE and analyzed by western blotting. Rad18 forms were visualized using anti-HA (5000X, GeneTex GTX21208) primary and anti-rabbit (10 000X, Thermo Scientific 31460) secondary antibodies, and PGK with anti-PGK (10 000X, Molecular Probes A6457) primary and anti-mouse (10 000X, Thermo Scientific 31430) secondary antibodies.

Denaturing Ni-nitriloacetic acid (Ni-NTA) chromatography was applied to detect *in vivo* PCNA ubiquitylation (Ulrich and Davies 2009). Whole cell extracts were prepared according to a protocol (Jiang *et al.* 2019). Briefly, 200–200 ml of cell cultures co-expressing 7His-PCNA and wt or mutant Rad18 were grown in SC-leu medium, since selection for the plasmid expressing the essential *POL30* gene is not necessary. At A_600_:1 half of the cultures were treated with 0.02% MMS for 90 minutes. Then cells were collected and washed, and resuspended in 1.5 ml of 0.3 M NaOH. After a 2 minutes incubation at RT, cells were collected and resuspended in 350 µl of lysis buffer [60 mM Tris/HCl pH 6.8, 5% glycerol, 2% SDS, and 4% 2-mercaptoethanol (ME)] and boiled at 100 °C for 8  minutes. Samples were clarified by centrifugation at 1,500 g for 20  minutes, and the supernatants were transferred into new tubes containing 1 ml of dilution buffer (10 mM Tris/HCl pH 8, 100 mM sodium-phosphate buffer pH 8, and 8 M urea), 30 µl of Ni-NTA beads, 15 mM imidazole and 0.05% Tween-20. Samples were incubated at 4 °C for 4 hours on a rotator and then washed three times with 1–1 ml of dilution buffer containing 0.05% Tween-20, and then three times with wash buffer (10 mM sodium-phosphate buffer pH 8, 500 mM NaCl, 0.5% NP-40, and 0.05% Tween-20). His-tagged PCNA was eluted and denatured by incubating the Ni-beads for 10  minutes at 60 °C in 30 µl of HU buffer (8 M urea, 200 mM Tris/HCl, pH 6.8, 1 mM EDTA, 5% SDS, 0.1% bromophenol blue, and 1.5% dithiothreitol). Samples were resolved on a 12% SDS gel and transferred onto a PVDF membrane (Immobilon-P, Merck Millipore Ltd.). After blotting, membranes were pre-treated either in 6 M guanidyl-HCl, 20 mM Tris/HCl pH 7.5, 5 mM 2-ME for 30  minutes at 4 °C for labeling with anti-Ub antibody (1500X, P4D1, Cell Signaling Technology), or 2 × 6  minutesat RT in stripping buffer (0.2 M glycine, 0.1% SDS, 1% Tween-20) for labeling with anti-PCNA antibody (1000X, Abcam 5E6/2). Anti-mouse (10 000X, Thermo Scientific 31430) and anti-rabbit (10 000X, Thermo Scientific 31460) secondary antibodies were used, respectively.

### Statistical analysis

Student’s*t*-test using Excel (Microsoft, Redmond, WA, USA) was applied to compare separate groups. *P*-values of <0.05 were considered statistically significant. * *P* < 0.05, ** *P* < 0.01, *** *P* < 0.001.

## Results

### Design of the Rad18 deletion mutants

**Figure 1:**
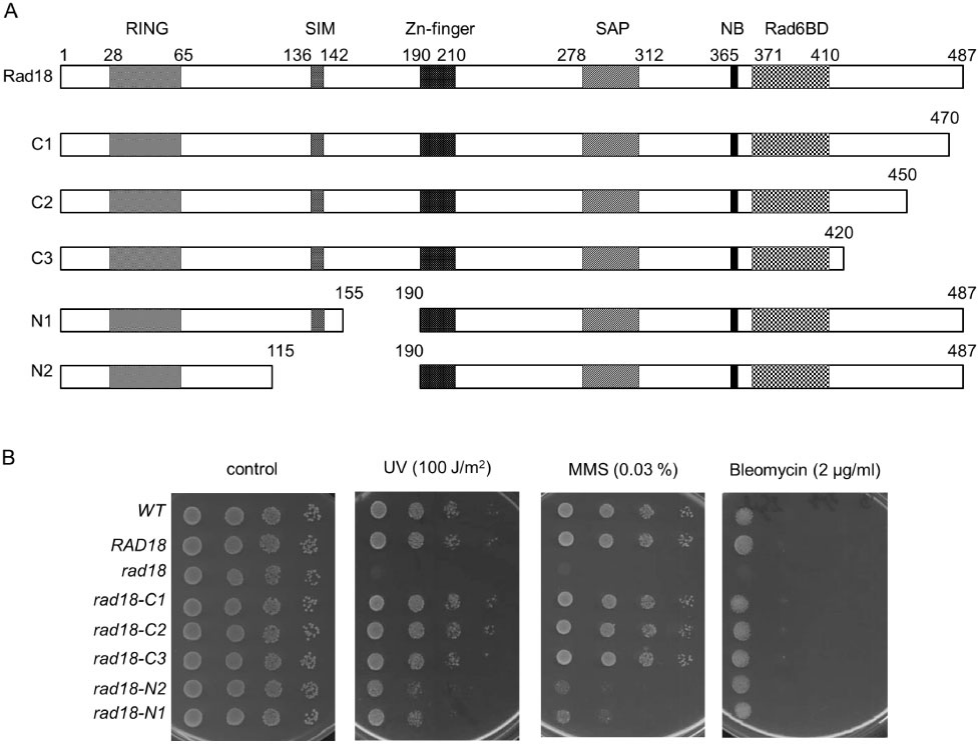
The C-terminal truncations do not affect Rad18 function. (A) Schematic representation of wt and deletion mutant Rad18 proteins engineered in this study. RING, RING finger domain; SIM, SUMO interaction motif; Zn-finger, Rad18-type Zinc finger domain; SAP, SAF-A/B, Acinus, and PIAS domain; NB, Walker-A nucleotide binding motif; Rad6BD, Rad6 binding domain. Amino acid numbers are marked above the schemes and the mutants are named on the left. (B) Sensitivity of the *rad18* strains to DNA damaging agents. Tenfold serial dilutions of the indicated strains were spotted on plates either exposed to the indicated UV dose, or containing the indicated amount of MMS or bleomycin. Several UV, MMS, and bleomycin doses were applied, but only plates with the most appropriate doses are presented. Genotypes are in italic, with the expressed Rad18 variants indicated.

To better understand the role of Rad18 in DDT, we aimed to investigate the contribution of extended, uncharacterized regions of the protein to its function. At the C-terminus, residues between 371 and 410 were shown to be necessary for binding Rad6 (Bailly *et al.*1997b). However, C-terminal to this Rad6 binding domain there is an acidic tail of 77 amino acids without any assigned function. Therefore, we created three overlapping truncations in this region, the longest eliminating almost the complete C-terminal sequence following the Rad6-binding domain. At the N-terminus, Rad18 has a RING domain characteristic of a group of E3 ubiquitin ligases and essential for the function of Rad18 in DDT, and a Zn-finger in the middle of the protein whose function is still not clear. We engineered two overlapping deletions between the RING and Zn-fingers with the bigger one removing more than half of the region between the two domains. The schematics of the wt Rad18 and the deletion mutants, referred to as C1, C2, C3, N1, N2, are shown in Figure 1A.

### The C-terminal truncations do not influence the *in vivo* function of Rad18 in DDT

The mutant proteins were ectopically expressed under the native *RAD18* promoter in haploid yeast cells, from which the genomic copy of *RAD18* was removed. The *in vivo* effects of the deletions were assessed by treating the cells with DNA damaging agents like ultraviolet (UV)light, the methylating agent MMS, and the radiation mimetic drug bleomycin. Surprisingly, as Figure 1B shows, all three C-terminal mutants displayed wt sensitivity to all three agents, even at high doses. These results suggested that the C-terminal acidic tail did not significantly contribute to the function of Rad18 in DNA damage response.

### The N-terminal deletions in Rad18 affect the Mms2-dependent sub-pathway

**Figure 2:**
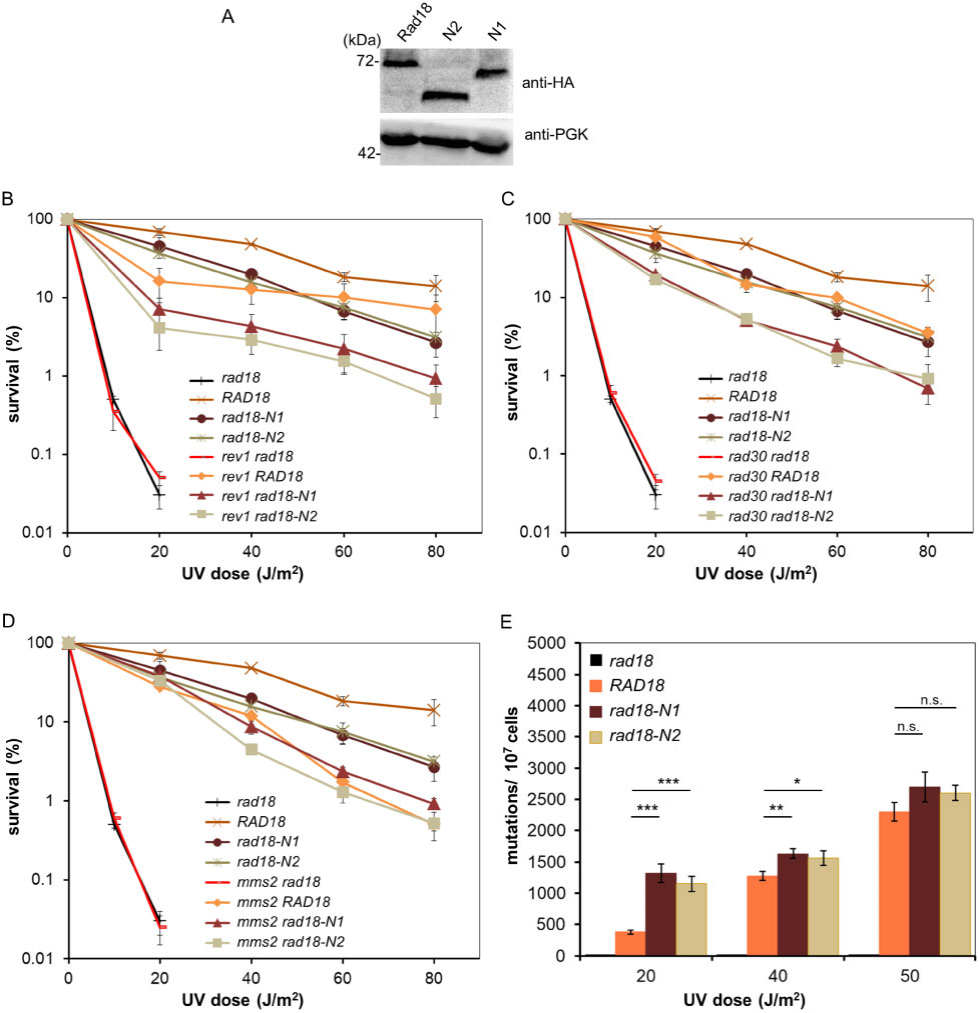
Genetic characterization of the N-terminal mutants. (A) Intracellular expression level of wt Rad18, N1, and N2 mutants. Whole cell extracts were prepared from strains expressing the C-terminally tagged proteins that were visualized with anti-HA antibody. PGK served as a loading control. (B–D) Epistasis analysis with the *REV1*, *RAD30*, and *MMS2* dependent sub-pathways. Quantitative analysis of UV survival of single and double mutants with (B) *rev1* (C) *rad30*, and (D) *mms2*. Results represent the averages of three experiments. Standard deviations are indicated. (E) UV-induced mutagenesis. Forward mutation rates at the *CAN1* locus were determined after exposing the cells to the indicated doses of UV. Averages of three experiments and standard deviations are shown. *P*-values representing the significance of difference are shown as *, *P* < 0.05; **, *P* < 0.01; ***, *P* < 0.001.

Contrary to the C-terminal truncations, the two N-terminal deletion mutants, N1 and N2, conferred almost identical mild sensitivities to yeasts upon UV and MMS, however, they did not influence the sensitivity to bleomycin (Figure 1B). First, we verified that the deletions did not significantly alter the expression level of *RAD18* resulting in the observed sensitivities. Therefore, we tagged the wt and mutant *RAD18* genes with three copies of the haemagglutinin tag (3HA) at the C-terminus, and examined their cellular expression level in whole cell extracts. As Figure 2A shows, both N1 and N2 displayed similar expression level as the wt Rad18 ruling out that the deletions affected the intracellular abundance of Rad18. Next, we investigated whether the increased sensitivities could be linked to selective inactivation of a sub-branch of the Rad18 governed DDT. For this reason, we separately inactivated the three sub-branches in the *rad18-N1* or *rad18-N2* strains by deleting a prominent gene of the given sub-branch, and investigated their genetic relations to the mutant *RAD18* genes by epistasis analysis. In these experiments, the N2 mutant conferred slightly higher sensitivities than the N1 mutant to different strains upon UV and MMS treatment. We surmised that this might be because the deletion in this mutant removed the SIM motif, as well. The analysis showed, that additional deletion of *REV1* or *RAD30* resulted in increased sensitivities over the single mutants suggesting that these sub-branches were functional in the *rad18-N1* and *rad18-N2* strains (Figure 2, B and C). In accordance, induced mutagenesis was active in these strains confirming that the N-terminal deletions in Rad18 did not disturb the mutagenic sub-pathway involving the TLS polymerases (Figure 2E). Moreover, at lower doses mutagenesis was higher in the mutants compared to the wt suggesting that the mutagenic TLS pathway gained more ground over the error-free pathway. At higher dose the rates were very similar probably due to the activation of additional, *RAD18* independent pathways like homologous recombination. Next, we examined the relationship of the *rad18* mutants to the third, error-free branch. We found, that upon deleting *MMS2* the resulting double mutant strains displayed similar sensitivities as the *mms2Δ* single mutant strain suggesting that the N-terminal deletions in *RAD18* affected the *MMS2*-dependent sub-pathway (Figure 2D).

### Amino acids 155-190 of Rad18 are necessary for Rad5 binding

**Figure 3:**
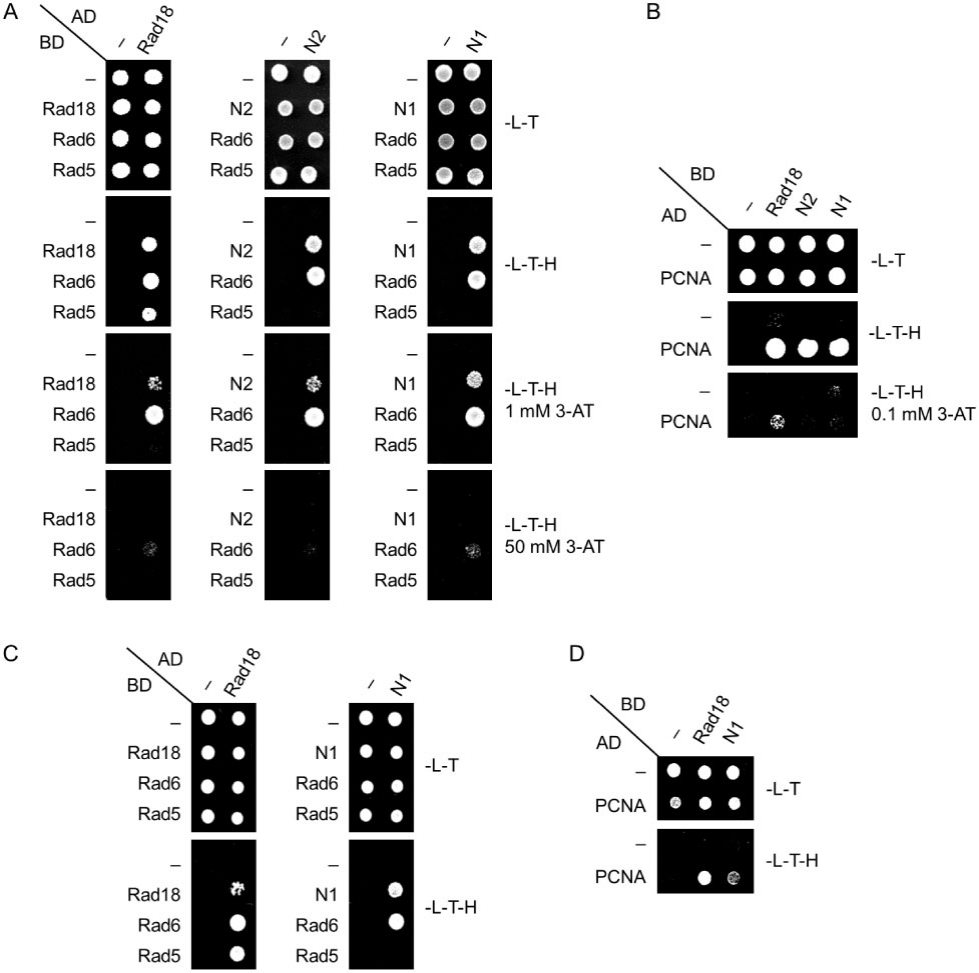
Formation of the Rad18-Rad5 complex is inhibited by the N-terminal deletions. The interactions of N1 and N2 were tested (A), (C) with Rad6, Rad5, and self, as well as (B), (D) with PCNA by yeast two-hybrid assay. Experiments were performed in (A) and (B) using wt PJ694A strain, while in (C) and (D) *rad18 pol30-K164R* strain. AD, fusion with Gal4 activation domain; BD, fusion with Gal4 DNA binding domain; -L-T, -Leu -Trp control medium; -L-T-H, -Leu-Trp-His test medium; 3-AT, 3-aminotriazol.

Rad5 together with Mms2 and Ubc13 acts in the error-free branch of DDT promoting template switch. Among these proteins, Rad5 has been shown to directly interact with Rad18 (Ulrich and Jentsch 2000). Therefore, we set out to investigate whether the N-terminal deletions in Rad18 inhibited the formation of the Rad18-Rad5 complex, by applying yeast two-hybrid assays. As controls, we included known binding partners of Rad18 in the experiments like Rad6, PCNA, and the Rad18 protein itself. The results showed that the wt Rad18, and also both N1, and N2, could form dimers with themselves and they could bind Rad6 and PCNA, too (Figure 3) The mutations did not detectably affect the strength of the binding either, except with PCNA where slight weakening of the interactions could be observed in the presence of 3-AT. (Figure 3B). However, contrary to the wt, neither N1, nor N2 showed interaction with Rad5 (Figure 3A). Similar experiments using a *rad18Δ* strain expressing K164R PCNA from the genomic locus corroborated that the detected interactions were direct and they were not mediated by the endogenous Rad18 or ubiquitylated PCNA (Figure 3, C and D). Taken together, these results suggested that the region in Rad18 between amino acids 155–190 missing from both N1 and N2 was necessary for Rad5 binding.

### The Zn-finger of Rad18 mediates the interaction with Rad5

**Figure 4:**
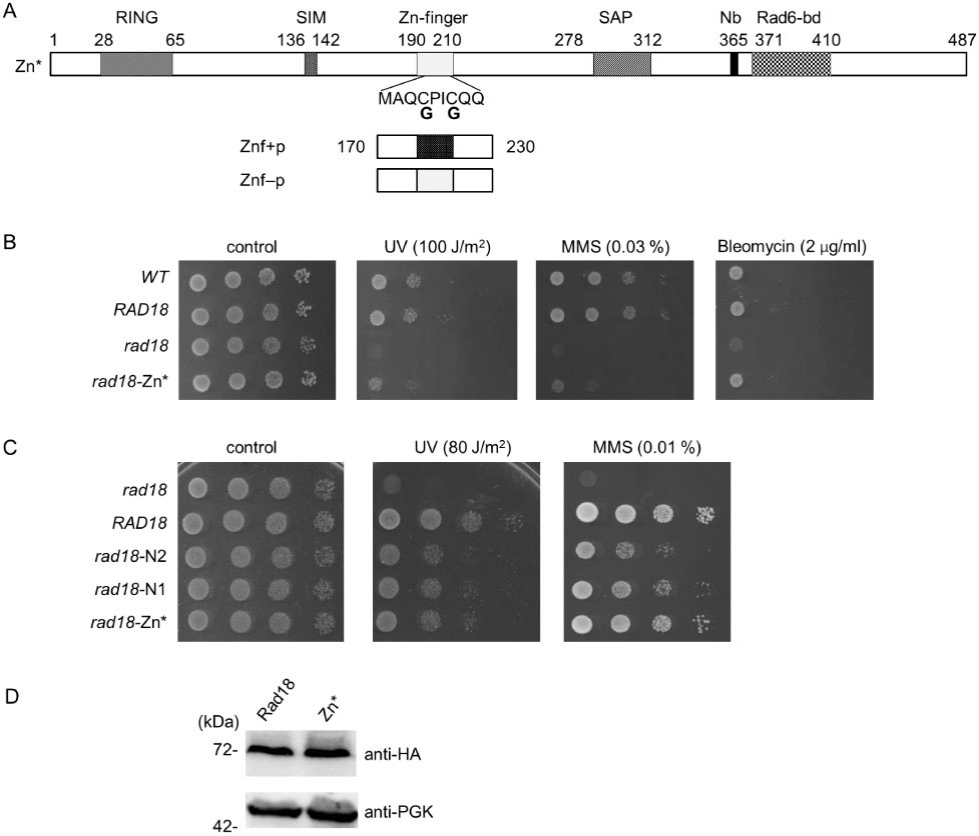
Characterization of the Zn-finger mutant Rad18. (A) Schematic structure of the Zn* mutant and the Znf+ and Znf- peptides. Domains designated as in Figure 1A. The positions of cysteines substituted to glycines are indicated. (B) Sensitivity of the rad18-Zn* strain to different DNA damaging agents. The assay was performed as described in Figure 1B. (C) Comparing the sensitivities of the *rad18-N1*, *rad18-N2*, and rad18-Zn* mutants upon UV and MMS. Tenfold serial dilutions of the indicated strains were spotted on plates either irradiated with UV or containing the indicated dose of MMS. (D) Equal expression of the wt Rad18 and the Zn* mutant. The experiment was performed as described for Figure 2A.

**Figure 5:**
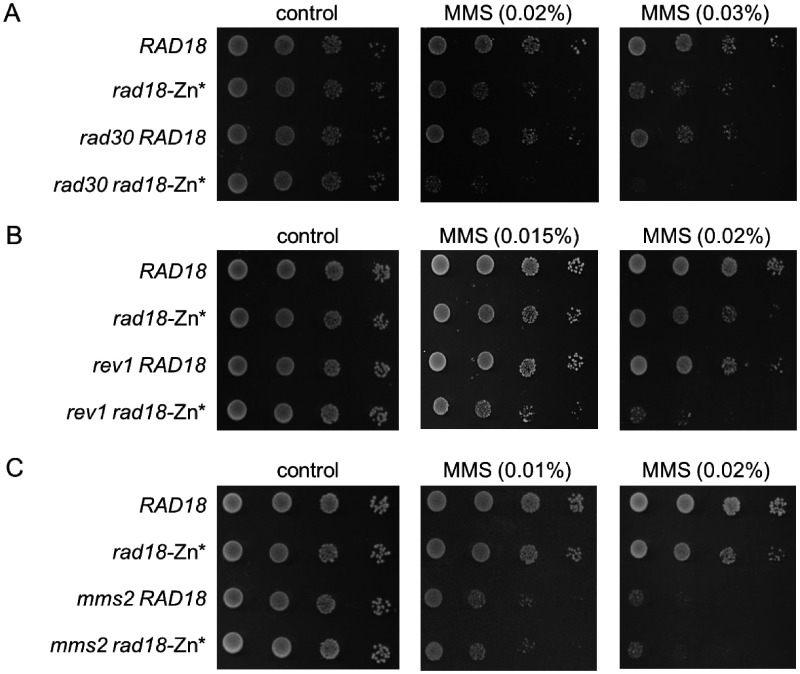
Epistasis analysis of the rad18-Zn* mutant. MMS sensitivities of the double mutants containing deletions of (A) *RAD30*, (B) *REV1*, and (C) *MMS2* were examined. Tenfold serial dilutions of the indicated strains were spotted on plates containing the indicated concentration of MMS.

As the Zn-finger is situated next to the deletions in the N1 and N2 proteins, we aimed to investigate whether it was also involved in mediating the association with Rad5. For this purpose, we disrupted the Zn-finger by replacing the N-terminal two zinc-coordinating cysteine residues of the Zn-finger with glycines (Figure 4A). The functionality of the resulting CC190,193GG Rad18 mutant protein, which we named Zn*, was checked in sensitivity assays. As it is shown in Figure 4B, the rad18-Zn* strain exhibited the same sensitivity pattern as the *rad18-N1* and *rad18-N2* strains: it showed mild sensitivities to UV and to MMS but none to bleomycin, compared to the wt strain. Next, we confirmed that the CC190,193GG mutations did not perturb the intracellular expression level of Rad18 (Figure 4D). Interestingly, the *rad18-N1*, *rad18-N2*, and rad18-Zn* strains exhibited very similar sensitivities to UV irradiation, however, upon MMS treatment the rad18-Zn* strain was more resistant (Figure 4C). Even so, when we examined the genetic relations of the Zn* mutant to the three sub-branches of the Ra6/Rad18 pathway, we got the same results as with the N1 and N2 mutants (Figure 5). Deletion of *RAD30* or *REV1* in the rad18-Zn* strain further sensitized the single mutants indicating that the function of the TLS polymerases was not affected by the *CC190,193GG rad18* mutations (Figure 5, A and B). Contrary to that, the mms2Δ rad18-Zn* strain was not more sensitive than the *mms2Δ* single deletion strain indicating that the CC190,193GG mutations in Rad18 disturbed the Mms2-dependent branch (Figure 5C). Taken together, the above data suggested that the mutations in the N1, N2, and Zn* proteins affected the same biological function of Rad18.

**Figure 6:**
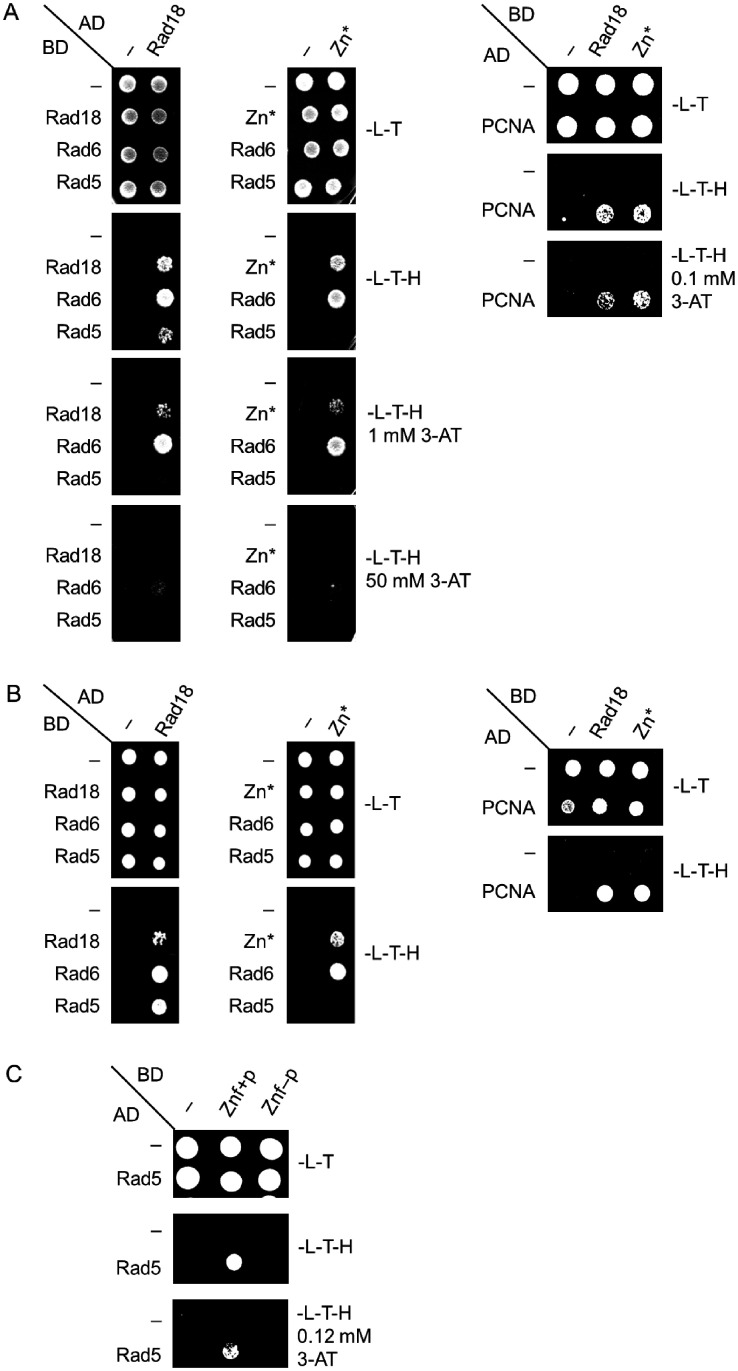
Yeast two-hybrid assay to test the interaction of the Zn* mutant, and the Znf+, and the Znf- peptides. (A) and (B) Interaction of the Zn* mutant with itself, with Rad6, with Rad5, and with PCNA. Experiments were performed in (A) with wt PJ694A strain, (B) with *rad18 pol30-K164R* strain. (C) Interaction between Rad5 and the Znf+ and Znf- peptides. Labels are as in Figure 3.

Therefore, we investigated the effect of the CC190,193GG mutations on the interaction between Rad18 and Rad5 by applying yeast two-hybrid assays. Indeed, the results of these experiments indicated that the absence of the two C residues prevented the formation of the Rad18-Rad5 complex (Figure 6, A and B). Nevertheless, self-dimerization or the interaction with Rad6 or PCNA was not affected. Based on these, we concluded that without the Zn-finger Rad18 was not able to bind Rad5.

To obtain direct evidence on the role of the Rad18 Zn-finger, we tested the Rad5-binding capability of a short Rad18 fragment spanning from aa. 170 to 230 that consisted the Zn-finger (Figure 4A), in yeast two-hybrid experiments. Figure 6C shows, that this Znf+ peptide interacted with Rad5, whereas the Znf− peptide having the CC190,193GG amino acid changes did not show interaction. These experiments confirmed that the Zn-finger of Rad18 mediated the association with Rad5.

### DNA damage induced PCNA polyubiquitylation occurs in *rad18-N1* and rad18-Zn* cells

**Figure 7:**
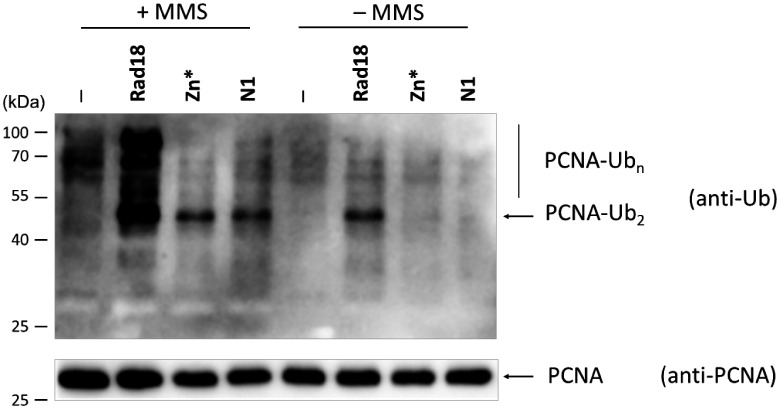
DNA damage induced PCNA polyubiquitylation is decreased in the *rad18-N1* and rad18-Zn* strains. The strains expressing the indicated Rad18 variants were untreated or treated with 0.02% MMS for 90 minutes. His-tagged PCNA from extracts prepared from 100 ml cultures were bound to Ni-beads, and ubiquitylated forms were visualized by Western blotting using anti-ubiquitin antibody (upper panel). PCNA was visualized on Ni-beads by anti-PCNA antibody (lower panel). The positions of di- and polyubiquitylated PCNA forms are labeled. Markers are shown on the left.

Formation of the Rad18-Rad5 complex is generally considered to be necessary for PCNA polyubiquitylation, hence to the functionality of the Rad5 branch. However, yeast strains expressing our Rad18 mutant proteins unable to interact with Rad5 displayed only mild sensitivities to UV and to MMS; they were less sensitive than the *mms2Δ* strain. This prompted us to check the activity of the Mms2 subpathway in the different strains by probing the DNA damage induced polyubiquitylation of PCNA. As Figure 7 shows, MMS treatment triggered PCNA polyubiquitylation in the wt and, though to a lesser extent, also in the *rad18-N1* and rad18-Zn* strains indicated by the appearance of bands corresponding to di- and polyubiquitylated forms of PCNA. The monoubiquitylated form could not be observed since the applied anti-ubiquitin antibody could not recognize it, as already reported by others (Hoege *et al.* 2002). Altogether, these findings raised the possibility that Rad5 could promote PCNA polyubiquitylation even without interacting with Rad18, though with a decreased efficiency.

### Spontaneous mutagenesis is affected in the N1, N2, and Zn* expressing strains

**Figure 8:**
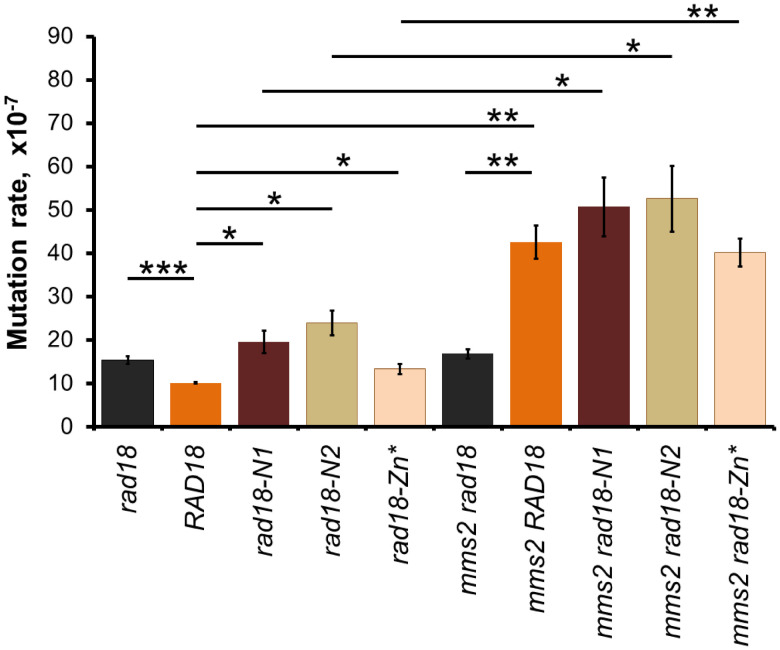
Spontaneous mutation rates are increased in the N1, N2, and Zn* expressing strains. Forward mutation rates at the *CAN1* locus were determined using 10 parallel cultures for each strain. Genotypes are indicated in italic. Averages of minimum five experiments and standard errors are shown. *P*-values representing the significance of difference are shown as *, *P* < 0.05; **, *P* < 0.01; ***, *P* < 0.001.

Besides DDT, another process that is influenced by Rad5, Mms2, and Ubc13 is spontaneous mutagenesis. In the lack of either Rad5, Mms2, or Ubc13, spontaneous mutation rate is highly elevated (Broomfield *et al.*1998; Liefshitz *et al.*1998; Brusky *et al.* 2000). We aimed to investigate, how the absence of interaction between Rad18 and Rad5 affected this process. As Figure 8 shows, a ∼2,5-1.5-fold raise in the rate of spontaneous mutations was observed in the *rad18-N1*, *rad18-N2*, and rad18-Zn* expressing strains, whereas a ∼4-fold increase was detected in the *mms2Δ* strain over the wt rate. Deletion of *MMS2* in the *rad18* mutant strains resulted in a spontaneous mutation rate similar to that measured in the *mms2Δ* single mutant indicating that the mutations were epistatic to *mms2Δ* in respect of spontaneous mutagenesis, as well.

## Discussion

This study identifies Zn-finger of yeast Rad18 as the Rad5 binding domain, and shows that its N-terminal neighboring sequence is also important for complex formation. By engineering internal deletions and point mutations in the full-length *RAD18* gene, we investigated the contribution of different regions of the protein to DNA damage bypass. Our yeast two-hybrid experiments show that mutations that abolish the conserved cysteine residues of the Rad18 Zn-finger, or remove its N-terminal nearby sequence prevent the interaction with Rad5. Our genetic data with the *RAD18* mutant strains are consistent with a defect in the Rad5 dependent error-free subpathway. However, these strains are considerably less sensitive to UV and to MMS treatments than the *mms2Δ* strain that is completely devoid of DNA damage induced PCNA polyubiquitylation. Also, spontaneous mutagenesis in the mutants increased to a smaller extent compared to the *mms2Δ* strain. These results suggested that DNA damage induced PCNA polyubiquitylation still occurred in the N1, N2, and Zn* expressing strains, which we could confirm in *in vivo* experiments . One possible explanation is that the Rad18-Rad5 complex is not absolutely required for PCNA polyubiquitylation. In its absence, probably the direct interaction between Rad5 and PCNA, reported earlier (Hoege *et al.* 2002), could promote a decreased rate of PCNA polyubiquitylation resulting in reduced activation of the error-free damage bypass pathway. Another possibility that we cannot exclude is that a weak interaction undetectable by yeast two-hybrid still takes place between the mutant Rad18 proteins and Rad5, which can support some level of PCNA polyubiquitylation.

**Figure 9:**
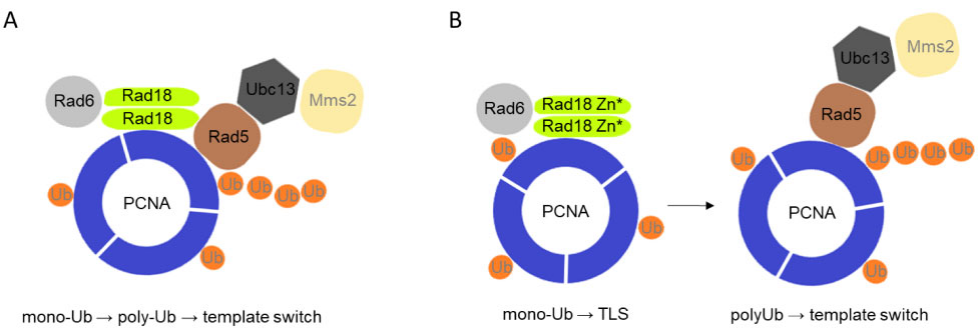
Suggested model of the effect of the Rad18-Rad5 interaction on PCNA ubiquitylation. (A) Monoubiquitylation and polyubiquitylation of PCNA is coupled in a single protein complex when Rad18 can interact with Rad5, giving preference to the error-free template switch pathway. (B) When Rad18 is not able to bind Rad5, the mono- and poly-ubiquitylation steps are carried out by two separate complexes and become uncoupled, and due to that the error-prone TLS pathway becomes prevalent.

There are conflicting results in the literature about the role of the Rad18 Zn-finger. Peptides containing the Zn-finger of human Rad18 were shown to bind ubiquitin, both *in vitro* and *in vivo* (Bish and Myers 2007; Notenboom *et al.* 2007; Huang *et al.* 2009; Rizzo *et al.* 2014). It was reported, that replacing one of the conserved cysteine residues (C207F) in the Zn-finger abolished homodimerization of human Rad18 *in vivo* (Miyase *et al.* 2005), whereas another study found that the same mutant could form homodimers *in vitro* (Notenboom *et al.*2007). Moreover, structural studies with human Rad18 identified the RING finger as the dimerization domain that mediates the assembly of an asymmetric complex containing two Rad18 and one Rad6 molecules (Huang *et al.* 2011; Masuda *et al.* 2012). Also, HLTF and SHPRH were both shown to bind human Rad18 via its Zn-finger, in a competitive manner with ubiquitin (Zeman *et al.* 2014). In the case of yeast Rad18, the N-terminal half of the protein containing the Zn-finger was shown to mediate both self-dimerization and Rad5 binding (Ulrich and Jentsch 2000). Our data indicate that, like its human homologue, yeast Rad18 binds Rad5 through its Zn-finger. However, as the Rad18 mutant proteins defective in Rad5 binding could still form homodimers, self-assembly must be mediated by other regions of the protein implying that Rad18 can bind itself and Rad5 at the same time. These results support the model that the two ubiquitin ligase-conjugase complexes, one composed of two Rad18 and one Rad6, and the other of Rad5-Mms2-Ubc13, could assemble into a single multiprotein complex (Figure 9). In such complex, PCNA mono- and polyubiquitylation would be spatially brought and linked together ensuring that once PCNA is monoubiquitylated, polyubiquitylation would follow, giving priority to the Rad5 mediated error-free branch over the mutagenic branch mediated by the TLS polymerases.

Our data also show, that neither the N-terminal deletions, nor the Zn* mutant confer additional sensitivity to bleomycin that causes DNA strand breaks. This is in agreement with previous results showing that even though both Rad18 and Rad5 play a role in the repair of DNA strand breaks, their functions in that process is independent from each other (Friedl *et al.* 2001). Unexpectedly, our experiments indicate that removing the last 67 residues from the C-terminal tail of Rad18 does not confer any sensitivity to UV, to MMS, or to bleomycin suggesting that the C-terminal tail of Rad18 does not have a significant role in DNA damage processing. Further experiments are needed to discover the function of this part of the protein, and to identify other structural elements mediating interactions between Rad18 and its numerous partners.

## Data availability

Strains and plasmids are available upon request. The authors affirm that all data necessary for confirming the conclusions of the article are present within the article, figures, and tables.
